# Abelson tyrosine kinase links PDGFbeta receptor activation to cytoskeletal regulation of NMDA receptors in CA1 hippocampal neurons

**DOI:** 10.1186/1756-6606-1-20

**Published:** 2008-12-12

**Authors:** Michael A Beazely, Manjula Weerapura, John F MacDonald

**Affiliations:** 1 Department of Physiology, University of Toronto, 1 King's College Circle, Toronto, ON M5S 1A8, Canada; 2 Department of Pharmacology, University of Toronto, 1 King's College Circle, Toronto, ON M5S 1A8, Canada; 3 The Robarts Research Institute, 100 Perth Drive, University of Western Ontario, London, ON N6A 5K8, Canada

## Abstract

**Background:**

We have previously demonstrated that PDGF receptor activation indirectly inhibits N-methyl-D-aspartate (NMDA) currents by modifying the cytoskeleton. PDGF receptor ligand is also neuroprotective in hippocampal slices and cultured neurons. PDGF receptors are tyrosine kinases that control a variety of signal transduction pathways including those mediated by PLCγ. In fibroblasts Src and another non-receptor tyrosine kinase, Abelson kinase (Abl), control PDGF receptor regulation of cytoskeletal dynamics. The mechanism whereby PDGF receptor regulates cytoskeletal dynamics in central neurons remains poorly understood.

**Results:**

Intracellular applications of active Abl, but not heat-inactivated Abl, decreased NMDA-evoked currents in isolated hippocampal neurons. This mimics the effects of PDGF receptor activation in these neurons. The Abl kinase inhibitor, STI571, blocked the inhibition of NMDA currents by Abl. We demonstrate that PDGF receptors can activate Abl kinase in hippocampal neurons via mechanisms similar to those observed previously in fibroblasts. Furthermore, PDGFβ receptor activation alters the subcellular localization of Abl. Abl kinase is linked to actin cytoskeletal dynamics in many systems. We show that the inhibition of NMDA receptor currents by Abl kinase is blocked by the inclusion of the Rho kinase inhibitor, Y-27632, and that activation of Abl correlates with an increase in ROCK tyrosine phosphorylation.

**Conclusion:**

This study demonstrates that PDGFβ receptors act via an interaction with Abl kinase and Rho kinase to regulated cytoskeletal regulation of NMDA receptor channels in CA1 pyramidal neurons.

## Background

Long-term potentiation (LTP) and long-term depression (LTD) are forms of plasticity which occur at the synapses between CA3 and CA1 pyramidal neurons of the hippocampus and they underlie some forms of spatial learning and memory [1,2]. Their induction is dependent upon the activation of N-methyl-D-aspartate (NMDA) receptors [3,4]. These receptors make a relatively minor contribution to the basal excitatory synaptic potential but they are recruited during high frequency stimulation when the depolarizations summate and cause a relief of their block my Mg^2+^. The NMDA receptor currents must be further enhanced during the high frequency stimulation by the calcium-dependent activation of Pyk2 and Src kinases in order to induce LTP [5]. Src is brought into proximity of the NMDA receptors through its binding to ND2, a protein which serves as a structural and morphological scaffold for the regulation of NMDA receptors by Src [6]. A variety of Gα q-coupled receptors stimulate phospholipase C (PLC) β and PKC and enhance NMDA receptors by targeting the Pyk2/Src regulation of NMDA receptors in CA1 neurons [7].

NMDA receptors bind directly to a variety of other scaffolding proteins including actin binding proteins [8]. Actin binding proteins link these receptors to the actin cytoskeleton and these protein-protein interactions are regulated by calmodulin binding and the influx of calcium via NMDA receptors such that the activity of these receptors is closely linked to actin cytoskeletal dynamics [8]. This actin anchoring may be permissive for the mechano-sensitivity of NMDA receptors [9] and is required for the inhibition of NMDA receptors by myosin light chain kinase and F-actin [10]. Therefore, actin cytoskeleton dynamics are pivotal to regulation of NMDA receptors.

LTD of the NMDA receptor-mediated component of the excitatory synaptic postsynaptic currents (epscs) is blocked by inhibition of serine-threonine phosphatases and can be prevented by using agents that stabilize the cytoskeleton in CA1 neurons [11]. We have previously shown that stimulation of the dopamine D2 receptor (a Gαi-coupled receptor) results in transactivation of platelet derived growth factor β (PDGFβ) receptors [12,13] and in turn PDGFβ receptor activation causes a long-lasting depression of NMDA receptor currents in CA1 neurons [12,14,15] that resembles the LTD of NMDA receptor epscs. For example, inhibition of NMDA receptor currents by the PDGFβ receptor is prevented by the serine-threonine phosphatase inhibitor calyculin A and is also dependent on the stability of the actin cytoskeleton [14,15]. The mechanism(s) whereby PDGFβ receptor signaling modifies the actin cytoskeleton in CA1 neurons to regulate NMDA receptor currents is unknown, however the inhibition does require activation of PLCγ and it is both calcium- and Src-dependent [14].

In a variety of cell types PDGFβ receptors form a signal complex with PLCγ, Src and a second tyrosine kinase, Abelson (Abl) kinase and downstream signaling is dependent upon interplay between of these enzymes [16]. Abl kinase is implicated in mechanisms of synaptic plasticity, plays a crucial role in growth cone motility, and is involved dendritic branching in developing neurons [17-20]. In Abl and Abl-related gene (Arg) double-knock out mice, the extent of dendritic branching in substantially reduced [20] and treatment of developing cultured neurons with the Abl inhibitor, STI571, decreases the complexity of neuronal dendritic branching via an increase in RhoA activity [21] and possible activation of Rho-associated kinase (ROCK) [22-24]. Despite the identification of Abl as an important regulator of several neuronal processes, it remains unclear how Abl is activated in neurons. In several systems, Abl kinases are also required for Rho-family GTPase coupling to the actin cytoskeleton and can reciprocally signal to ROCK [25]. Given the signaling connection between PDGFβ receptors and Abl as well as the evidence of Abl kinase regulation of neuronal signaling and development, we examined the possibility that in CA1 neurons Abl kinase is activated downstream of PDGFβ receptors and that Abl acting with ROCK is responsible for the alterations in actin cytoskeleton that underlie long-term depression of NMDA receptor activity.

## Results

### Abl kinase inhibits NMDA-evoked currents

**Figure 1:**
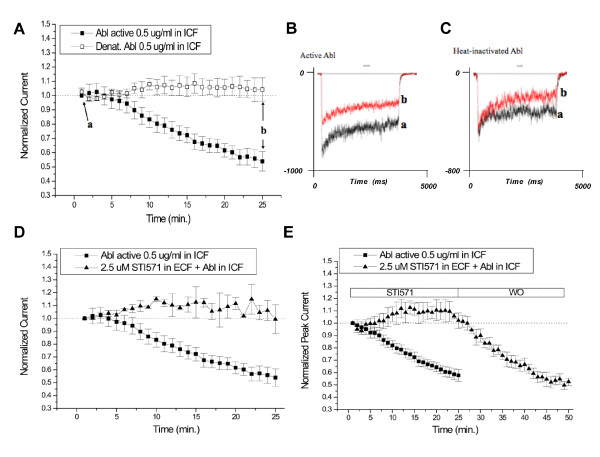
**Intracellular application of Abl decreases NMDA-evoked currents**. A) Active (black square) or heat-inactivated (denat., white square) Abl kinase (0.5 μg/mL) was included in the intracellular solution. NMDA-evoked currents were recorded once every minute and peak currents were normalized to the average of the first five currents recorded. At 25 minutes (b) Abl significantly reduced NMDA-evoked currents (p < 0.05, Student's t-test, n = 6). B, C) Representative currents from currents recorded in the presence of active Abl kinase or heat-inactivated kinase at t = 3 minutes (a) or t = 25 minutes (b). D) NMDA-evoked currents were recorded with active Abl kinase applied intracellularly in the ICF in the absence (black square) or presence (black triangle) of the Abl kinase inhibitor, STI571, applied in the ECF bath (n = 6). E) NMDA-evoked currents were recorded with active Abl kinase applied intracellularly in the ICF in the absence (black square) or presence (black triangle) of the Abl kinase inhibitor, STI571, applied in the ECF bath for 25 minutes (STI571), followed by a 25 minute washout period (WO) during which control ECF solution was applied (n = 6).

To examine the effects of Abl on NMDA currents, we acutely dissociated CA1 neurons from 2–3 week old Wistar rats. Whole cell NMDA currents were evoked by applying 50 μM NMDA and 0.5 μM glycine for 2 seconds every minute. Intracellular application of 0.5 μg/mL active Abl kinase resulted in a time-dependent depression of NMDA currents (Figure 1A). After 25 minutes, NMDA currents were reduced by 53 ± 5% (Figure 1A, B). In contrast, the inclusion of heat-inactivated Abl kinase in the patch pipette did not depress NMDA currents such that peak currents were maintained for the 25 minutes of recording (104 ± 8%, Figure 1A, C).

Chromosomal translocations involving Abl kinase result in a constitutively active kinase that causes chronic myeloid leukemia [26]. Recently, STI571 (imatinib mesylate, Gleevec™) has been used to successfully treat patients with this form of leukemia. STI571 is a potent inhibitor of Abl kinase as well as PDGFβ receptors with an IC_50 _value for Abl in the low micromolar range [27,28]. Active Abl kinase was introduced into the cell via the patch pipette whereas STI571 was added to the ECF. STI571 (2.5 μM) completely blocked the inhibition of NMDA currents by Abl kinase (Figure 1D). The inhibition of NMDA currents by Abl returned upon the washout of STI571 from the ECF (Figure 1E). These data show that STI571 inhibits the depression of NMDA currents induced by Abl and that this inhibition is reversible.

### PDGF receptors activate Abl in the hippocampus

**Figure 2:**
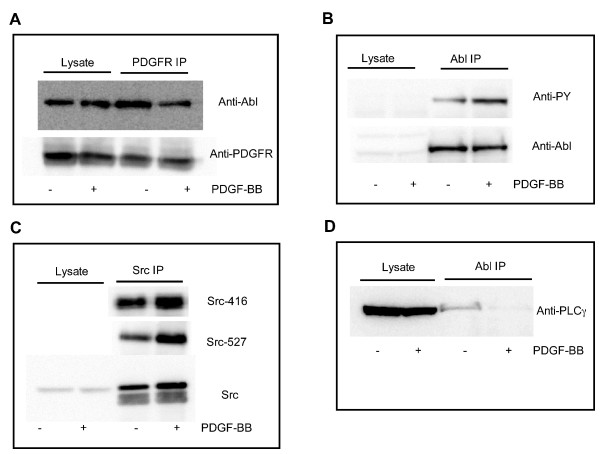
**PDGF receptors activate Abl kinase in hippocampal neurons**. Acutely dissected hippocampal CA1 slices were incubated in the absence or presence of 10 ng/mL PDGF-BB for 10 minutes. A) Slices were homogenized and the lysate was incubated with anti-PDGFβ receptor antibodies precoupled to agarose beads. Total lysate and immunoprecipitated samples were immunoblotted with anti-Abl (top) or anti-PDGFβ receptor (bottom). Blots are representative of 7 experiments. B) Slices were homogenized and the lysates were immunoprecipitated with an anti-Abl antibody. Lysates and immunoprecipitates were resolved via SDS-Page electrophoresis and immunoblotted with anti-phospho-tyrosine (PY) or anti-Abl kinase antibodies. Blots are representative of 7 experiments. C) Anti-Src antibodies were used to immunoprecipitated Src after treatment of slices with 10 ng/mL PDGF-BB for 10 minutes. Immunoprecipitated samples were separated by SDS-electrophoresis and membranes were probed with anti-tyrosine 146 (Src-416), anti-tyrosine-527 (Src-527), or anti-Src (Src) antibodies. Blots are representative of 4–6 experiments. D) Lysates were immunoprecipitated with anti-Abl antibodies and membranes were probed with anti-PLCγ antibodies. Blot is representative of 5 experiments.

Multiple signaling pathways including those initiated by PDGFβ receptors can activate the cytoplasmic pool of Abl kinase. To investigate the possibility that PDGFβ receptor activation could activate Abl kinase in hippocampal neurons, we incubated acutely dissected CA1 hippocampal slices with the PDGFβ receptor ligand, PDGF-BB. We then incubated CA1 hippocampal cell lysates with immobilized anti-PDGFβ receptor antibodies to examine whether PDGFβ receptors and Abl kinase physically associate in hippocampal neurons. Abl kinase was immunoprecipitated with PDGFβ receptors from hippocampal neurons and this association was significantly diminished upon PDGF-BB treatment of the hippocampal slices (Figure 2A, Abl immunoreactivity in PDGF receptor antibody pull-down was 0.65 ± 0.12 fold vs. control, n = 7, p < 0.05, unpaired Student's t-test). This observation supports the interpretation that Abl associates with PDGFβ receptors and is released upon activation of the receptor. To further examine the ability of PDGFβ receptors to activate Abl kinase in hippocampal neurons, we monitored the phosphorylation state of Abl kinase after incubation of hippocampal slices with PDGF-BB. Treatment with PDGF-BB for 10 minutes enhanced the tyrosine phosphorylation of Abl kinase (Figure 2B, 4.2 ± 0.8 fold vs. control, n = 7, p < 0.05 unpaired Student's t-test). These data show that treatment of CA1 hippocampal slices results in an increase in Abl tyrosine kinase phosphorylation and a change in the association of Abl kinase with the PDGF receptor.

We have previously demonstrated that both Src kinase and PLCγ are required for the inhibition of NMDA-evoked currents by PDGFβ receptors [14,29]. PDGFβ receptor activation activates Abl in HEK293 cells in a Src- and PLCγ-dependent manner [30,31]. PDGF-BB treatment of hippocampal slices enhanced the phosphorylation of both the tyrosine 416 (associated with increased Src activity) and tyrosine 527 (associated with decreased Src activity (Figure 2C) [32,33]. Consistent with previous studies examining the activation of Abl kinase, CA1 lysates immunoprecipitated with anti-Abl antibodies showed a decrease in the interactions of Abl with PLCγ (Figure 2D) and Src (not shown). Taken together, these results suggest that Abl interacts with PDGFβ receptors, PLCγ, and Src in hippocampal neurons and activation of PDGFβ receptors decreases the association of Abl with these interacting partners as well as causing an increase in the tyrosine phosphorylation of Abl kinase.

### PDGF receptor activation decreases the cytoplasmic pool of Abl kinase

**Figure 3:**
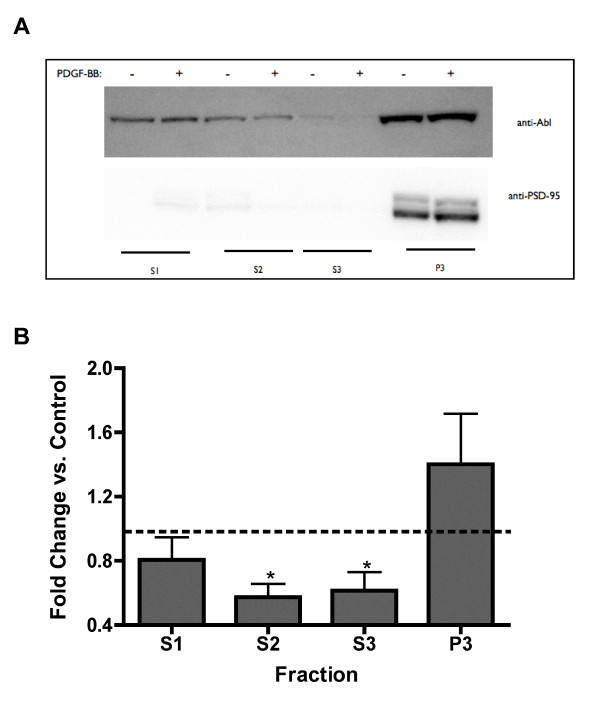
**Abl kinase immunoreactivity is decreased in the S2 and S3 fractions after PDGFβ receptor activation**. A) Hippocampal slices were treated for 10 minutes with 10 ng/mL PDGF-BB, were homogenized, and S2, S3, and P3 fractions were prepared as described in the methods section. Equal amounts of total protein from each fraction were resolved on SDS gels, transferred to nitrocellulose membranes, and immunoblotted with anti-Abl or anti-PSD-95 antibodies (n = 6). B) PDGF-BB treated fractions were normalized to control for each fractionation sample. Data represent the mean +/- standard error for 6 independent experiments. PDGF-BB treatment significantly decreased Abl kinase immunoreactivity in the S2 and S3 fractions, * p < 0.05, Student's unpaired t-test.

Abl kinase is localized in both the synaptosomal and post-synaptic density fractions in adult rat brain homogenates [34] and Abl immunoreactivity is detected pre- and post-synaptically [18]. Upon stimulation of hippocampal slices with PDGF-BB, Abl kinase immunoreactivity decreased in both the S2 (cytosolic) and S3 (light membrane) fractions (Figure 3A, B). There was a trend towards an increase in immunoreactivity for Abl kinase in the triton-insoluble fraction (Figure 3A, B), suggesting that Abl kinase is moving from the cytosolic and light membrane fraction to the triton-insoluble fraction. Conversely, it is possible that Abl is being rapidly degraded in the S2 and S3 fractions. The NR1 subunit of the NMDA receptor was not significantly altered with respect to subcellular localization (Table 1).

**Table 1:** The effect of PDGF-BB on the subcellular fractionation of PDGFβ receptors, ROCK, and NR1

Protein	Change in localization after PDGF-BB treatment			
	S2	S3	P3	

PDFGβ receptor	*n/a*	63 ± 15%^*a*^	136 ± 31%	

ROCK	60 ± 12%^*a*^	66 ± 30%	125 ± 30%	

NR1	*n/a*	81 ± 36%	130 ± 30%	

Hippocampal slices were treated for 10 minutes with 10 ng/mL PDGF-BB, were homogenized, and S2, S3, and P3 fractions were prepared as described in the methods section. Equal amounts of total protein from each fraction were resolved on SDS gels, transferred to nitrocellulose membranes, and immunoblotted with anti- PDFGβ receptor (n = 4), anti-ROCK (n = 3), or anti-NR1 (n = 3) antibodies. Data represent the mean +/- standard error, *a *p < 0.05, Student's unpaired t-test.

### Abl inhibition of NMDA currents is blocked by the ROCK inhibitor Y-27632

**Figure 4:**
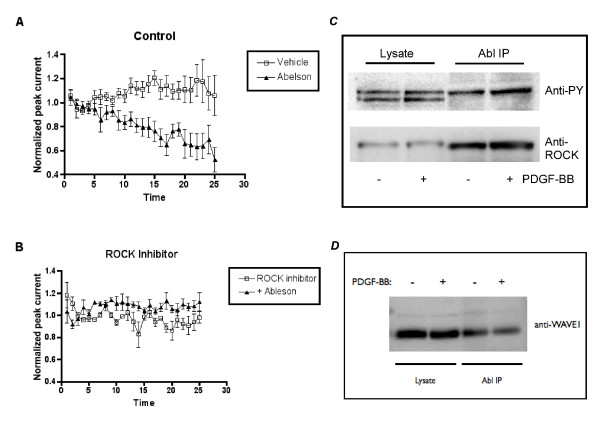
**The ROCK inhibitor, Y-27632, attenuates Abl kinase-induced inhibition of NMDA evoked currents**. A) Abl kinase (black triangle) or control (white square) was included in the intracellular solution. NMDA-evoked currents were recorded once every minute and peak currents were normalized to the average of the first five currents recorded (n = 7).). At 25 minutes Abl significantly reduced NMDA-evoked currents (p < 0.05, Student's t-test, n = 5), B) NMDA-evoked peak currents were recorded every minute in the presence of 1 μM Y-27632 with control (ROCK inhibitor, white square) or Abl kinase (+Abelson, black triangle) included in the intracellular solution. Peak currents were normalized to the average of the first five currents recorded (n = 5). C) Slices were homogenized and the lysates were immunoprecipitated with an anti-ROCK antibody. Lysates and immunoprecipitates were resolved via SDS-Page electrophoresis and immunoblotted with anti-phospho-tyrosine (PY) or anti-ROCK antibodies. Blots are representative of 4 experiments. D) Slices were homogenized and the lysates were immunoprecipitated with an anti-Abl antibody. Lysates and immunoprecipitates were resolved via SDS-Page electrophoresis and immunoblotted with anti-WAVE1 antibody. Blot is representative of 4 experiments.

Both PDGFβ receptors and Abl kinase are involved in cytoskeletal dynamics in multiple systems [19,35,36]. Incubation of acutely dissociated hippocampal neurons with the Rho kinase (ROCK) inhibitor, Y-27632 (IC_50 _~ 0.3 μM) [37], attenuated the ability of intracellularly-applied Abl kinase to inhibit NMDA currents (Figure 4A, B). PDGF-BB also enhanced the tyrosine phosphorylation of ROCK by 1.87 +/- 0.25 (Figure 4C, p < 0.05, Student's unpaired t-test). Furthermore, ROCK underwent a similar change in subcellular localization as Abl kinase (Table 1). Abelson kinase is also associated with actin cytoskeletal dynamics via its association with WAVE1. Interestingly, Abl associates with WAVE1 in hippocampal neurons and treatment of hippocampal slices with PDGF-BB decreased the association of Abl with WAVE1 (Figure 4D) [38]. The ability of the ROCK inhibitor to block the Abl inhibition of NMDA currents implies that the inhibition of NMDA currents may be dependent on changes Abl-mediated alterations in the actin cytoskeleton.

## Discussion

In the central nervous system, Abl kinase is involved with several crucial physiological processes. However the majority of studies have used transgenic deletions of Abl and/or Arg or small molecule inhibitors of the kinases [20,21]. Mechanisms of Abl regulation by cell surface receptors and intracellular signaling pathways in neurons remain unclear, and the role of Abl in post-synaptic neuronal signaling, specifically in NMDA receptor regulation, is unknown. We demonstrate that active Abl kinase inhibits NMDA currents in isolated hippocampal neurons. The inhibition of NMDA currents by Abl is completely and reversibly blocked by STI571, an Abl kinase (and PDGFβ receptor kinase) inhibitor. Although Abl and PDGFβ receptors may be inhibited by STI571, we have not previously observed a significant basal activity level of PDGF receptors in hippocampal neurons in previously published reports [12,13] and unpublished observations.

Several lines of evidence link cell surface receptors to the activation of Abl kinase. The most detailed studies on the regulation of Abl involve the activation of Abl by PDGFβ receptors in fibroblasts [30,31]. In hippocampal neurons, Abl appears to be regulated by similar mechanisms. Abl associates with PDGFβ receptors, PLCγ, and Src in CA1 hippocampal neurons. Upon treatment of acutely isolated hippocampal slices with PDGF-BB, the association of Abl with each of these proteins is decreased and this coincides with an increase in tyrosine phosphorylation of Abl kinase.

Interestingly, PDGF-BB treatment increased the phosphorylation of Src at both tyrosine 416 (tyrosine phosphorylation here potentiates Src activity) and 527 (tyrosine phosphorylation inhibits Src activity) [39]. Activation of Src kinase via the phosphorylation of tyrosine 416 is associated with a potentiation of NMDA receptor currents and an increase in long-term potentiation [40-42]. However, we have previously demonstrated that application of PDGF-BB inhibits NMDA currents and that this *inhibition *is Src-dependent [14]. The identification of Abl as a downstream target in hippocampal neurons coupled with the requirement of Src to fully activate Abl after PDGF receptor activation may help reconcile the apparent contradiction in the requirement of Src for an inhibition of NMDA receptor currents by PDGF receptor activation and the direct potentiation of NMDA receptors by Src [14]. Recent work by Veracini et al. have identified least two distinct subcellular pools of Src mediating distinct signaling pathways in NIH 3T3 cells [43,44]. The increases in tyrosine phosphorylation at both the inhibitory and activation tyrosine residues of Src in the hippocampus suggest the intriguing possibility that two or more pools of Src may be present in neurons as well. One pool interacting with the PDGFβ receptor and leading to a PLCγ/Abl/Src complex that signals to the cytoskeleton via ROCK (with a resulting inhibition of NMDA receptors) and the other pool interacting directly with ND2 and the NMDA receptor complex to enhance NMDA receptor activity during LTP.

**Figure 5:**
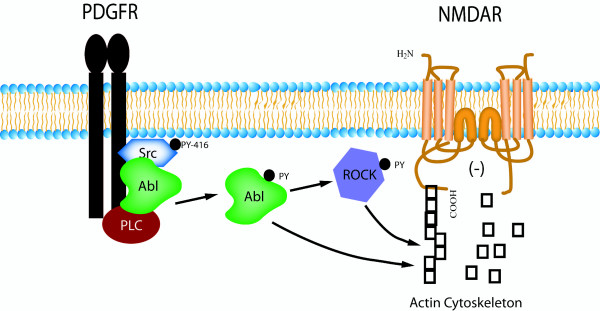
**Abelson kinase associates with PDGFβ receptors and inhibits NMDA receptor currents in a ROCK-dependent manner**. Abelson kinase (Abl) physically associates with PDGFβ receptors, PLCγ, and Src in hippocampal neurons. Upon activation of PDGFβ receptors, Abelson kinase is tyrosine-phosphorylated and dissociates from the receptor. Introduction of Abelson kinase into isolated hippocampal neurons robustly inhibits NMDA receptor currents in a ROCK-dependent manner, possibly through changes in the actin cytoskeleton.

In cortical neurons, Abl and Arg are required for normal dendritic branching and are activated downstream of integrin receptors [17,20]. The regulation of dentritic spines and synapses are also important for synaptic plasticity in mature neurons, and depends heavily on actin cytoskeleton dynamics [45,46]. In contrast to previous reports suggesting ROCK activity is inversely proportional to Abl activity [23,47,48], treatment of hippocampal slices with PDGF-BB increased ROCK tyrosine phosphorylation. Furthermore, the ROCK inhibitor attenuated Abl-induced decreases in NMDA-evoked currents. These observations show that ROCK is required for Abl-mediated changes in the actin cytoskeleton that leads to inhibition of NMDA currents and that Abl-mediated inhibition of NMDA receptor currents requires RhoGTPase and ROCK signaling (Figure 5). Therefore, in addition to being crucial for neuronal development, Abl kinase regulates NMDA function in mature neurons and links PDGFβ receptor signaling to the actin cytoskeleton. Our results place Abl at an important intersection between alterations in the neuronal cytoskeleton and regulation of NMDA receptors.

## Conclusion

PDGF-BB treatment decreased the association of Abl with PDGFβ receptors. Furthermore, PDGF-BB treatment reduced the association of Abl with PLCγ and Src, suggesting that Abl is activated in hippocampal neurons in a manner similar as previously reported in non-neuronal cells. PDGF-BB treatment of hippocampal slices also induced a translocation of Abl from the cytosolic and membrane fraction to the triton-insoluble fraction. Previous studies have suggested that PDGFβ receptor activation ultimately alters NMDA receptor signaling by inducing changes in cytoskeletal dynamics and Abl kinase is linked in several systems to actin dynamics. Similar to extracellular treatment of neurons with PDGF-BB, the inclusion of Abelson kinase in the patch pipette robustly inhibited NMDA-evoked currents in isolated CA1 neurons and this inhibition was blocked by treatment of neurons with the Abl inhibitor STI571 and the ROCK inhibitor, Y-27632. These results suggest that PDGFβ receptors activate Abl kinase in hippocampal neurons and that Abl links PDGFβ receptor signaling to changes in the neuronal cytoskeleton that ultimately lead to changes in NMDA receptor signaling.

## Methods

### Reagents and antibodies

Active Abl kinase (mouse) was purchased from Upstate Biotechnology/Millipore (Belerica, MA). Each lot of active Abl kinase was tested for phosphorylation of a peptide substrate in an *in vitro *kinase assay (by the manufacturer). Briefly, each lot of Abl kinase had a specific activity (~1750 units/mg) where 1 unit is defined as incorporating 1 nmol phosphate into 50 μM Abltide (EAIYAAPFAKKK) per minute at 30°C in the presence of 100 μM ATP. PDGF-BB was purchased from R&D Systems (Minneapolis, MN, USA). Y-27632 was purchased from Calbiochem (San Diego, CA). NMDA, glycine, and all other chemical reagents were purchased from Sigma (St. Louis, MO, USA). Antibodies used include those raised against PDGFβ receptor (Epitomics, California, USA), Abelson kinase (Sigma), Src (Cell Signaling, Danvers, MDA) and phospho-Y416 and Y527 Src (Biosource, Carlsbad, CA), and phospho-tyrosine antibody (Santa Cruz, La Jolla, CA), and PLCγ, ROCK, and WAV1 (Cell signaling). For the immunoprecipitation of PDGFβ receptors, an agarose-conjugated anti-PDGFβR antibody from Santa Cruz was employed.

### Cell isolation and whole-cell recording

CA1 neurons were isolated from hippocampal slices of postnatal day 14–21 Wistar rats as previously described [49]. The extracellular solution was composed of 140 mM NaCl, 1.3 mM CaCl2, 25 mM N-2-Hydroxyethylpiperazine-N'-thanesulfonic acid (HEPES), 33 mM glucose, 5.4 mM KCl, and 0.5 μM tetrodotoxin, and 0.5 μM glycine, with pH of 7.3–7.4 and osmolarity ranging from 320–330 mOsm. Recordings were done at room temperature. The membrane potential was held at -60 mV throughout the recordings and with a voltage step of 10 mV was applied prior to NMDA application to monitor series resistance. Recordings were rejected if a series resistance change greater than 10% was observed. The intracellular solution consisted of 11 mM Ethyleneglycol-bis-(α-amino-ethyl ether) N,N'-tetra-acetic acid (EGTA) as intracellular calcium chelating buffer, 10 mM HEPES, 2 mM MgCl2, 2 mM Tetraethyl ammonium chloride (TEA-Cl) to block K+ channel, 1 mM CaCl2, 140 mM CsF, and 4 mM K2ATP. NMDA currents were evoked by rapid application of NMDA solution delivered from a multi-barreled fast perfusion system for 2 seconds in every minute. The solution was delivered at a rate of approximately 1 ml per minute. Abl or heat-inactivated Abl (0.5 μg/mL) was added to the ICF.

### Immunoprecipitation and western blotting

The CA1 region of the hippocampus was microdissected from day 14–22 Wistar rat pups. CA1 slices (5–10 per condition) were incubated for 10 minutes with vehicle or PDGF-BB. Slices were washed in ice-cold ECF and homogenized in solubilization buffer (20 mM Tris, pH = 7.5, 150 mM NaCl, 1 mM EDTA, 1 mM EGTA, 30 mM Na pyrophosphate, 1 mM βglycerophosphate, 1% Triton (1% NP-40 was substituted for immunoprecipitation), 1 mM Na3VO4, and MINI cocktail (Roche, Mannheim, Germany). Samples were centrifuged at 14,000 × g for 20 minutes and lysates were used for protein determination. Total protein concentration was determined by BCA Protein Assay (Pierce, Rockford, Il, USA). For Western blot, equal amounts of protein were loaded for each sample. For immunoprecipitations, lysate protein concentrations were normalized and lysates were incubated with primary antibody and protein A/G beads (Sigma) overnight at 4°C. Beads were washed three times and boiled in SDS loading buffer for 5 minutes before separation by SDS page electrophoresis. Proteins were transferred to nitrocellulose membranes, blocked with 5% non-fat dry milk in Tris-buffered saline for 1 hr at room temperature or overnight at 4°C, and incubated in primary antibodies for 1 hr or overnight at 4°C. Membranes were washed three times in Tris-buffered saline with 0.1% Tween-20, incubated with HRP-conjugated secondary antibodies for 1 hr, washed again, and bound antibodies were visualized by the enhanced chemiluminescence method. Densitometric analysis of Western blots was performed using the Kodak Image Station 2000R software.

### Subcellular fractionation

Hippocampal tissue was incubated with vehicle or PDGF-BB and the tissue was dounce homogenized in fractionation buffer 1 to yield the S1 fraction (buffer 1: 0.32 M sucrose, 4 mM HEPES, pH 7.3, MINI protease inhibitor cocktail 1:10, and 1 mM Na3VO4). The homogenized tissue was centrifuged at 1000 × g for 10 minutes at 4 degrees. The supernatant was retained and centrifuged at 14,000 × g for 20 minutes. The supernatant (S2) was retained and the insoluble material was resuspended in fractionation buffer 2 (buffer 1 + 1% triton). The resuspended mixture was centrifuged at 100,000 × g for 60 minutes and the supernatant (S3, triton-soluble membrane fraction) was retained. The pellet (P3) was resuspended and dissolved in fractionation buffer 3 (buffer 1 + 0.5% SDS + 1% deoxycholate). Protein concentration was determined by BCA protein assay and lysates were subjected to Western blotting as described above.

### Statistical analysis

Statistical analysis of the data was completed using Prism^® ^GraphPad program. Graphs and sample tracings were made from Origin^® ^program. All data was reported as mean ± SEM. Significance level was set at α = 0.05. Data was analyzed by Student's t-test or unpaired Student's t-test where appropriate.

### Animals

All animal experiments were performed in agreement with the guidelines of the policies on the Use of Animal at the University of Toronto.

## Abbreviations

NMDA: N-methyl-D-aspartate; PLC: phospholipase C; PDGF: platelet-derived growth factor; ROCK: Rho-associated kinase

## Competing interests

The authors declare that they have no competing interests.

## Authors' contributions

MAB carried out the biochemical experimentation, contributed to electrophysiogical recording, and drafted the manuscript. MW carried out the electrophysiological recording. JFM participated in the design and coordination of the study and helped to draft the study
